# Supplementary material for: Altered Conformational Landscape upon Sensing Guanine Nucleotides in a Disease Mutant of Elongation Factor-like 1 (EFL1) GTPase
Source: Biomolecules. 2022 Aug 19;12(8):1141. doi: 10.3390/biom12081141 (PMC9405973; doi:10.3390/biom12081141)
Supplement: Supplementary file 1 [file biomolecules-12-01141-s001.zip › biomolecules-1847364-supplementary.pdf]

Supplementary material

Supplementary Figures

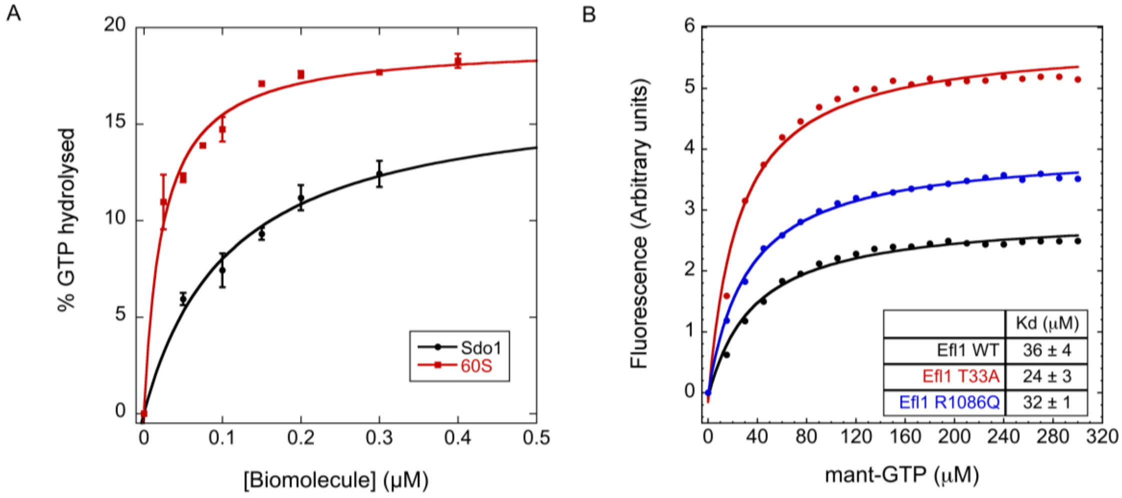

Supplementary Figure S1. Active complex formed with different constructs of Efl1 together with the effector protein Sdo1 and the ribosomal subunit 60S. (A) Representative curves of the percentage of GTP hydrolysed by 0.1 µM Efl1 R0186Q in the presence of increasing concentrations of Sdo1 (black trace) and 0.1 µM Efl1 R0186Q, 0.3 µM Sdo1 with increasing concentrations of 60S ribosomal subunits (red trace). Each data corresponds to the average and standard deviation of three independent measurements. (B) Binding of mant-GTP to different constructs of Efl1 in the presence of Sdo1 and 60S subunits. Emission of the mant-GTP at 440 nm was plotted as a function of nucleotide concentration. Solid traces represent the fit of the data to a one binding site model.

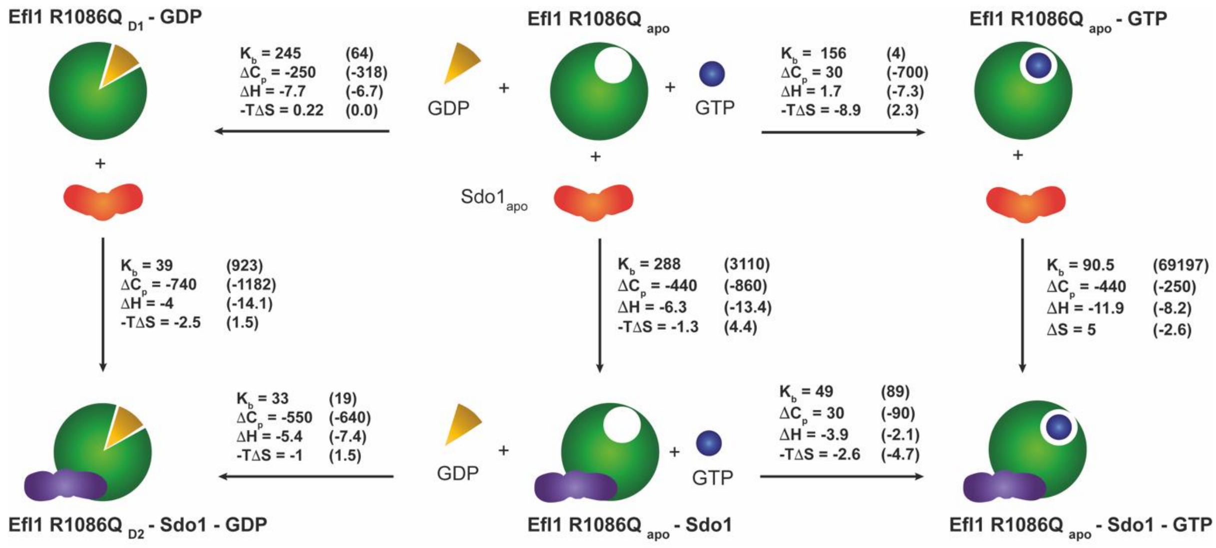

Supplementary Figure S2. **Energetics of the coupled equilibria between mutant Efl1 R1086Q, Sdo1 and guanine nucleotides in the absence of magnesium ions at 30°C.** Analysis indicates that mutant Efl1 adopts three different conformations: the *apo* conformation of the enzyme adopted irrespective if it is free or bound to GTP and/or Sdo1 (Efl1 $R1086Q_{apo}$) and a D-like conformation bound to GDP alone (Efl1 $R0186Q_{D1}$) that undergoes a conformational change upon binding to Sdo1 (Efl1 $R0186Q_{D2}$). Data in parenthesis corresponds to that of the wild-type Efl1 as reported in [3]. Units: K_b – mM^{-1}, -TΔS and ΔH – kcal mol^{-1}, ΔCp_b – cal mol^{-1} K^{-1}.

Supplementary tables

Supplementary Table S1. Summary of kinetic data for the activities of Efl1 and mutants alone and in complex with the effector Sdo1.

	k_{cat} (min^{-1})	K_m (µM)	k_{cat} / K_m (M^{-1} s^{-1})
Efl1 R1086Q	0.67 ± 0.03	146 ± 22	76 ± 12
Efl1 L910K	0.58 ± 0.03	185 ± 30	52 ± 9
Efl1 WT	0.67 ± 0.02	155 ± 2	72 ± 10
Efl1 R1086Q • Sdo1	0.67 ± 0.02	84 ± 7.5	131 ± 11
Efl1 L910K • Sdo1	0.75 ± 0.03	90 ± 9	140 ± 15
Efl1 WT • Sdo1	0.64 ± 0.01	53 ± 3	200 ± 11

Supplementary methods

Enzyme kinetics

Hydrolysis of GTP was measured using the Enzchek Phosphate Assay kit (Molecular Probes) as described by Webb [1]. Standard reactions consisted of 100 µL containing 6 µM of Efl1 R1086Q and Efl1 L910K, 0.3 mM MESG, 1.0 U purine nucleoside phosphorylase (PNP) and the indicated amounts of GTP. Measurements done in the presence of effector protein contained 25 µM of Sdo1. The reaction buffer consisted of 50 mM HEPES-KOH pH 7.5, 150 mM NaCl, 5 mM $MgCl_2$. Reactions were initiated and the GTPase was added after 3 min. Time courses were followed by recording the change in absorbance at 360 nm in a Cary 60 UV-Vis spectrophotometer (Agilent Technologies). Background absorbance was recorded prior to the addition of the GTPase and subtracted from the sample signals. The slope of the measured time courses corresponded to the GTP hydrolysis rate. The concentration of released phosphate was estimated using a molar absorption coefficient at 360 nm of 14,386 M^{-1} cm^{-1} previously described in [2]. Data were analysed by nonlinear regression to the Michaelis-Menten equation with the program Origin Version 5.0 (OriginLab, Co., Northampton, MA).

Assembly of the Efl1•Sdo1•60S complex

To ensure measuring the binding of Efl1 to guanine nucleotides when present in complex with Sdo1 and the 60S subunits, we first established the molar ratios needed of each biomolecule to bias the experimental set up. We reasoned that monitoring the GTPase activity would report on binding occupancy of Efl1 to the 60S subunit. Hydrolysis of GTP mediated by Efl1 R0186Q in the presence of different amounts of Sdo1 and 60S subunits was measured as described above. Initially, 0.1 µM of Efl1 R1086Q was titrated with increasing concentrations of Sdo1 (0.1 – 0.5 µM), activity approached a maximum as the concentration of Sdo1 corresponded to 0.3 µM suggesting that Efl1 had reached saturation. A second titration varying the concentrations of 60S subunits in a mixture of 0.1 µM Efl1 R1086Q and 0.3 µM Sdo1 was set up in the same conditions.

Steady state fluorescence measurements

The experiments were carried out at 25 °C in a buffer consisting of 0.1 M potassium acetate, 0.2 M HEPES-KOH pH 7.4, 2.5 mM magnesium acetate, 50 mM sucrose, 2 mM DTT, 0.1 mM PMSF, 0.1 mM benzamidine, 8 mM putrescine, 1 mM spermidine. Samples consisted of 0.25 µM GTPase, 0.7 µM Sdo1 and 0.3 µM 60S ribosomal subunits. Dissociation constants were measured for the interaction between the fluorescent guanine nucleotide analogues mant-GTP (Jena Bioscience), and GTPase•SBDS•60S. Binding was monitored by recording the fluorescence enhancement of the mant-nucleotides after excitation at 355 nm. Dilution at the end of each titration was no longer than 2.5% of the initial volume. Data were corrected for the background signal by subtracting the fluorescence of the same ligand concentration without the GTPase in the reaction mixture. Maximum emission at 440 nm was replotted as a function of nucleotide concentration and analysed

by nonlinear regression to a single-site binding model with the program Origin Version 5.0 (Origin Lab, Co., Northampton, MA).

1. Webb, M.R. A continuous spectrophotometric assay for inorganic phosphate and for measuring phosphate release kinetics in biological systems. *Proc Natl Acad Sci U S A* **1992**, *89*, 4884-4887, doi:10.1073/pnas.89.11.4884.
2. Gijsbers, A.; García-Márquez, A.; Luviano, A.; Sánchez-Puig, N. Guanine nucleotide exchange in the ribosomal GTPase EFL1 is modulated by the protein mutated in the Shwachman-Diamond Syndrome. *Biochem Biophys Res Commun* **2013**, *437*, 349-354, doi:10.1016/j.bbrc.2013.06.077.
3. Luviano, A.; Cruz-Castañeda, R.; Sánchez-Puig, N.; García-Hernández, E. Cooperative energetic effects elicited by the yeast Shwachman-Diamond syndrome protein (Sdo1) and guanine nucleotides modulate the complex conformational landscape of the elongation factor-like 1 (Efl1) GTPase. *Biophys. Chem.* **2019**, *247*, 13-24, doi:10.1016/j.bpc.2019.02.003.
